# An Oral 3D Printed PLGA-Tocopherol PEG Succinate Nanocomposite Hydrogel for High-Dose Methotrexate Delivery in Maintenance Chemotherapy

**DOI:** 10.3390/biomedicines10071470

**Published:** 2022-06-22

**Authors:** Pierre P. D. Kondiah, Thankhoe A. Rants’o, Sifiso S. Makhathini, Sipho Mdanda, Yahya E. Choonara

**Affiliations:** Wits Advanced Drug Delivery Platform Research Unit, Department of Pharmacy and Pharmacology, School of Therapeutic Sciences, Faculty of Health Sciences, University of the Witwatersrand, Johannesburg, 7 York Road, Parktown, Johannesburg 2193, South Africa; pierre.kondiah@wits.ac.za (P.P.D.K.); thankhoe.rantso@wits.ac.za (T.A.R.); sifiso.makhathini@wits.ac.za (S.S.M.); sipho.mdandah7@gmail.com (S.M.)

**Keywords:** 3D printed systems, cancer therapy, methotrexate, drug delivery, nanocomposites, biodegradable

## Abstract

High-dose methotrexate (HDMTX) is one of the chemotherapeutic agents used to treat a variety of cancers in both adults and children. However, the toxicity associated with HDMTX has resulted in the spread of infections and treatment interruption. Further, poor bioavailability due to efflux pump activities mediated by P-glycoprotein has also been linked to poor therapeutic effects of methotrexate following oral administrations. D-α-Tocopheryl poly-ethylene glycol 1000 succinate (TPGS) is known to improve the bioavailability of poorly soluble drugs by inhibiting P-gp efflux activities, thus enhancing cellular uptake. Therefore, to achieve improved bioavailability for MTX, this study aimed to design and develop a novel drug delivery system employing TPGS and a biodegradable polymer, i.e., PLGA, to construct methotrexate-loaded nanoparticles fixated in alginate-gelatine 3D printable hydrogel ink to form a solid 3D printed tablet for oral delivery. The results indicated that high accuracy (>95%) of the 3D printed tablets was achieved using a 25 G needle. In vitro, drug release profiles were investigated at pH 1.2 and pH 7.4 to simulate the gastrointestinal environment. The in vitro release profile displayed a controlled and prolonged release of methotrexate over 24 h. The in silico modeling study displayed P-gp ATPase inhibition, suggesting enhanced MTX absorption from the gastrointestinal site. The 3D-printed hydrogel-based tablet has the potential to overcome the chemotherapeutic challenges that are experienced with conventional therapies.

## 1. Introduction

Methotrexate (MTX) is a commonly used chemotherapeutic drug for different types of cancer including childhood acute lymphocytic leukemia (ALL) and autoimmune disease [1,2]. A high dose (≥500 mg/m^2^) of MTX (HDMTX) is used to achieve an effective plasma concentration in the target organs to treat several adult and pediatric malignancies, including ALL, osteosarcoma, and lymphomas [3,4,5]. MTX inhibits cancer cell growth by blocking the enzyme dihydrofolate reductase (DHFR), necessary for DNA synthesis [3]. However, variable pharmacokinetics profile intricate dosing schedules compromise the drugs’ bioavailability and therapeutic efficacy [6,7]. As an alternative, clinicians have resorted to the use of intramuscular injections of HDMTX per week, which can negatively impact patients’ compliance [8]. The development of drug delivery systems (DDS) continues to demonstrate hope in the field of medicine as the alternative to addressing therapeutic challenges associated with the use of conventional HDMTX formulation for cancer therapy and other disease conditions [1].

NPs have been shown to achieve superior tumor targeting, improve drug bioavailability, and increase cellular uptake efficiency through surface functionalization [9,10]. While oral drug administration is considered the most desirable route of administration due to its convenience and high tolerance by patients, especially in the case of chronic diseases that require frequent administration [11,12]. However, poor aqueous solubility and bioavailability, enzymatic stability, gastrointestinal absorption, and susceptibility to the efflux pumps in the gastrointestinal tract (GIT) remain the key challenges in achieving the desired therapeutic effect upon oral drug administration [13]. In addition, cancer cells can upregulate P-glycoprotein (P-gp) expression in order to resist chemotherapy-induced cell death. P-gp acts as the primary efflux pump for most anticancer drug molecules, becoming the main contributing factor to the multidrug-resistant phenotype in cancer by reducing the accumulation of chemotherapeutics after oral administration [14]. To date, no effective multi-drug resistance (MDR) treatment approach is available for clinical use to reverse the activity of P-gp [14,15]. However, d-α-tocopheryl polyethylene glycol 1000 succinate (TPGS) has demonstrated some ability to overcome the effects of P-gp through ATPase inhibition [16]. TPGS is an amphiphilic molecule derived from vitamin E and polyethylene glycol (PEG) 1000 [17,18]. Over the years, researchers have demonstrated that the extensive use of TPGS in drug delivery can be attributed to its biological and physicochemical properties such as high biocompatibility, enhanced solubility, enhanced permeation, and selective antitumor activity [19,20]. Hence, more nanoformulation systems modified with TPGS have been shown to improve the efficacy of orally delivered anticancer drugs through inhibition of drug efflux by TPGS 1000, along with enhancing cellular uptake.

Biodegradable poly (lactic-co-glycolic acid) (PLGA) is a well-known biocompatible synthetic co-polymer and it is one of the extensively researched polymers as a drug carrier due to its ability to encapsulate hydrophobic and hydrophilic drugs [21,22]. PLGA nanoparticles (NPs) have demonstrated the ability to improve the antitumor activity of anticancer drugs by increasing bioavailability through protection against bio and chemical degradation, enhancing intracellular penetration, and drug absorption [23,24]. Hence, PLGA has been widely employed as a stable and effective delivery carrier for a range of cancer-targeting treatments of diverse dimensions [22]. Despite the effectiveness of PLGA NPs in overcoming the inherent limitations of systemic drug delivery [25,26]. However, due to the hydrophobic nature of PLGA, they can be easily eliminated via the reticuloendothelial system (RES). To overcome this challenge, PLGA NPs have been decorated through surface modification with a range of polymers such as TPGS, chitosan, and poloxamers to evade possible elimination by the internal biological system [27]. 

In addition, RES elimination, transport across the intestinal epithelium remains the most critical barrier to achieving efficient oral delivery. The extreme acidic conditions within the gastric region (pH 1–2.5) are also another factor that significantly reduces the bioavailability and efficacy of the drug via chemical degradation [28]. However, several studies have shown that the use of NPs incorporated in hydrogels may be able to withstand the harsh acidity to preserve sensitive drugs [29]. However, one of the drawbacks of using hydrogels such as alginates is the large pore sizes and low mechanical strength that often result in burst release in regions of extreme pH due to protonation and relaxation of crosslinked chains [30]. Such limitations in the use of hydrogels reduce the ability to provide controlled and sustained drug release over extended periods [31]. One approach to precisely controlling the design process is via the use of 3D-(bio)printing. Recently, there has been a growing interest in fabricating 3D (bio)printed drug delivery systems due to their advantages for personalized medicine [32].

The 3D (bio)printing is a specialized additive manufacturing approach for producing aqueous-based hydrogels such as alginate-gelatine composites. Using this method will allow more robust control of the porosity and mechanical strength of the hydrogel to control the release of embedded drug-loaded NPs. Studies have shown that modifications of pore density and structure can alter diffusivity pathways for superior control of drug release [32]. Alginate and gelatine form a liquefied gel at room temperature and thus can be used as a bio-ink at low temperatures. Incorporated temperature-sensitive anticancer drugs can be stably printed using alginate and gelatinous bio-inks without destroying drug activity [33,34]. According to a study by Luo and co-workers (2018), a sodium alginate-gelatine (SA-GL) bio-ink was developed using 3D-(bio)printing with a temperature-controlled printing platform. Gelatine-based hydrogels are well suited for 3D (bio)printing and hence were selected in this study as the primary polymeric framework due to their proven thermolability, biocompatibility, biodegradability, non-immunogenicity, and structural integrity [35,36]. To benefit from the individual properties of sodium alginate and gelatine, these were co-fabricated to form a liquefied hydrogel and rendered suitable as a bio-ink for 3D (bio)printing as an approach to control the physicochemical design parameters and subsequent drug release from embedded TPGS-PLGA NPs. We propose the design of TPGS modified PLGA NPs loaded with the model anticancer drug methotrexate (MTX) with the aim of improving the efficiency and the release profile of high-dose MTX (HDMTX). 

Therefore, to overcome the poor oral bioavailability of HDMTX, this study explored the synthesis of TPGS-PLGA NPs that were embedded within a 3D-(bio)printed alginate-gelatin hydrogel matrix. The 3D (bio)printing was used to have superior control over the system design parameters via physicochemical and physicomechanical modifications, leading to a controlled release of HDMTX after oral administration. Although MTX has an oral bioavailability in the region of 64–90%, this decreases significantly at oral doses >25 mg due to saturation of the carrier-mediated transport of MTX [6]. Dose-dependent GIT absorption is one of the major concerns as a mechanism for the low bioavailability of oral MTX tablets when used as an anticancer agent. Therefore, the use of NPs as the delivery vehicle will promote a better tissue penetration of HDMTX through the extracellular space as studies have shown that drug distribution in tissue may be limited [37,38]. Besides being a P-gp inhibitor, the inclusion of TPGS in the formulation of PLGA NPs has demonstrated high cellular uptake, which can significantly improve the adsorption/permeation of drugs with poor bioavailability [39,40].

## 2. Materials and Methods

### 2.1. Materials

D-α-Tocopherol polyethylene glycol 1000 succinate (TPGS1000), Poly (D, L-lactide-co-glycolide) (PLGA), methotrexate (MTX), distilled water, HPLC grade acetone, ethanol and acetonitrile, mannitol were purchased from Sigma (Sigma-Aldrich, St. Louis, MO, USA). A simulated gastrointestinal environment (at buffer pH 1.2 and 7.4) was prepared from analytical grade reagents according to the method reported by Sardo et al., 2019 [41]. All of the reagents procured were of the highest analytical grade. Alginic acid sodium salt from brown algae (sodium alginate), methotrexate, phosphate-buffered saline, hydrochloric acid, gelatine powder, and calcium chloride dihydrate were purchased from Sigma (Sigma-Aldrich, St. Louis, MO, USA). All of the reagents procured were of the highest analytical grade. 

### 2.2. Phase 1: Synthesis of Methotrexate-Loaded Tocopheryl Polyethylene Glycol Succinate-Functionalized Polylactide-Co-Glycolic Acid Nanoparticles

#### 2.2.1. Preparation of Methotrexate-Loaded TPGS-PLGA Nanoparticulate System

The MTX-loaded TPGS-functionalized PLGA NPs were prepared using a modified nanoprecipitation technique adapted from Cerqueira and co-workers [42]. Briefly, 200 mL of deionized water was mixed with ethanol (80 mL) to prepare a 40% *w*/*v* ethanol-aqueous solution. TPGS (2 mL) was then dissolved in 100 mL of the ethanol-aqueous solution using continuous magnetic stirring at 90 °C for 2 h until TPGS was completely dissolved. After that 50 mg of MTX was dissolved in 100 mL of the TPGS solution using a magnetic stirrer set at 30 °C for 20 min. A PLGA acetonitrile mixture was prepared separately by dissolving PLGA (700 mg) in acetonitrile (100 mL). The PLGA solution was then added dropwise (1 mL/min) in a volume ratio of 1:10 to the TPGS solution under gentle magnetic stirring for 20 min at 35 °C to allow the self-assembly of the NPs. The excess organic solvent was removed by evaporation using a heated magnetic stirrer set at 80 °C for 2 h under a fume hood. The nanoparticle solution was then filtered thrice using a 0.45 μm Millipore syringe filter and immediately lyophilized and stored at 2 °C. 

#### 2.2.2. Componential Analysis of Chemical Structure Integrity Post Nanoparticle Formation

In order to assess the chemical integrity of native and combined components of the MTX-loaded TPGS-functionalized PLGA NPs, Fourier-Transform Infra-Red (FTIR) spectroscopy (PerkinElmer Spectrum 100, Llantrisant, Wales, UK) was used to identify and characterize the pharmaceutical stability of the MTX, TPGS, and PLGA in their native and combined state [43]. Moreover, this technique was used to determine the impact on the chemical stability of loading MTX in the PLGA NPs and to observe any possible significant changes in functional groups. The FTIR spectra were recorded at 20 °C ranging from 500 to 4000 cm^−1^ for samples of MTX, PLGA, and the MTX-loaded TPGS-functionalized PLGA NPs. 

#### 2.2.3. Nanometric Characterization of the MTX-Loaded TPGS-Functionalized PLGA Nanoparticles

Using various techniques, nanometric characterization was undertaken to determine the surface morphology, particle shape, size, density, and surface energy. A Zetasizer NanoZS (Malvern Instruments Ltd., Malvern, Worcestershire, UK) was used to determine the particle size and zeta potential of the NPs. The NPs were dispersed in deionized water, filtered (0.22 μm filter MilliporeSigma, Burlington, MA 01803, USA), and placed into disposal cuvettes (for size measurement) or capillary cells (for zeta potential determination). The viscosity and refractive index of the continuous phase were set to those specific to deionized water. To obtain a size distribution and zeta potential profile, measurements were taken in triplicate (*n* = 3) for each sample. 

#### 2.2.4. Morphology Characterization of MTX-Loaded TPGS-PLGA Nanoparticles

The nanoparticle morphology was examined using scanning electron microscopy (SEM). Powdered samples of the nanoparticles were placed onto an aluminum specimen stub covered with a double-sided carbon adhesive disc and sputter-coated with both palladium and gold for 4 min at 20 KV. SEM images of the methotrexate-loaded TPGS-PLGA nanoparticles samples were viewed using an SEM (SIGMA VP, Zeiss Electron Microscopy, Carl Zeiss Microscopy Ltd.; Cambridge, UK). 

#### 2.2.5. Thermal Analysis of Methotrexate-Loaded TPGS and PLGA Nanoparticles

To characterize the thermal behavior of methotrexate-loaded TPGS-PLGA nanoparticles, TGA (PerkinElmer STA 6000, Beaconsfield, UK) and a Mettler Toledo, DSC1, STARe Instrument (Schwerzenbach, Switzerland) were used, respectively. The analysis was performed for each reactant, drug, unloaded, and loaded nanoformulation sample. For TGA, a 5–10 mg sample was carefully weighed and placed in a crucible. Prior to analysis, standard temperature mode was selected, and samples were tested in the range of 30–900 °C with a 10 °C/min temperature rise and 20 mL/min rate of nitrogen purging. In total, 5–10 mg of each sample was carefully weighed and sealed in an aluminum crucible for DSC. Standard temperature mode was selected, the baseline was optimized, and each sample was tested at the −10–300 °C with a 10 °C/min temperature rise and 20 mL/min rate of nitrogen purging.

#### 2.2.6. In Vitro Release Evaluation of Methotrexate from the TPGS-PLGA Nanoparticles

To investigate the in vitro release of MTX from the TPGS-PLGA nanoparticles, Methotrexate-loaded TPGS and PLGA nanoparticles (*n* = 3) were incubated in 30 mL of PBS and positioned in an orbital shaking incubator at 37 °C for 24 h. The release of MTX was determined at different pH conditions 1.2 and 7.4 to mimic the stomach pH and intestinal pH, respectively. Sampling occurred at a specified time interval (5, 30, 60, 90, 120, 180, 240, 300, 360, 420, 480, and 720 min); however, stomach release simulation was conducted for up to 120 min. The 1 mL samples were collected as per time intervals and a fresh buffer solution was added to maintain sink condition. Samples were centrifuged at 5000× *g* for 10 min to allow nanoparticles to settle into pellets. Supernatants were then analyzed for UV absorption of MTX at 303 nm using the Nanophotometer UV/Vis spectrophotometer NP80 (Implen, München, Germany).

### 2.3. In Silico Analysis of P-gp Inhibition by TPGS

P-gp is one of the adenosine triphosphate (ATP)-binding cassette (ABC) transporter proteins and, at the gastrointestinal tract site, it is expressed in the apical site of the gastrointestinal (GI) mucosa [44,45,46]. This protein has two coordinated homologous halves each with a transmembrane domain (TMD) and a cytosolic nucleotide-binding domain (NBD). The TMD is made of six transmembrane helices that form a large hydrophobic cavity that has a capacity to bind a broad range of substrates molecules while the NDB binds and hydrolyses ATP [47,48,49]. TPGS is a non-ionic surfactant with a hydrophilic polar polyethylene glycol (PEG) chain and a lipophilic α-tocopherol head joined by a succinate linker. Interestingly, TPGS is known to inhibit the ATPase activity of P-glycoprotein [16,50,51]. Though TPGS has been shown to inhibit P-gp in cellular studies [16,52,53], the molecular interaction of TPGS and P-gp has not been intensely explored. Using homology modeling to generate human P-gp 3D structure, Liu et al. [51] investigated the possible binding profiles of TPGS through molecular dynamics simulations. Recently, various electron microscopy and X-ray diffraction studies optimized the human P-gp 3D structure in various states such as inward-facing where NBDs are far apart, the transition state, and outward-facing conformation where the NBDs are closer to each other [49,54,55,56]. P-gp undergoes these conformational changes during the catalytic cycle process [57]. The outward-facing conformation of P-gp is reported to efficiently model the substrate efflux capability and this transporter assumes this state upon ATP binding [54]. Given this, the current study, for the first time, assessed the TPGS binding potential on the outward-facing 3D structure of P-gp (PDB ID: 6C0V). Furthermore, given that TPGS inhibits the ATPase activity of P-gp and in accordance with previous studies [50,51], one ATP-binding site was chosen for receptor grid generation and TPGS docking. 

Schrödinger Release 2018-2 docking suite, Maestro version 11.6, (Schrödinger LLC, New York, NY, USA) was used for the docking assay. This molecular modeling program has been effectively used in the docking of polymers and surfactants [58,59,60]. The polymeric structures of PEG and TPGS were built through the Maestro Polymer Builder tool. For both PEG and TPGS, PEG was selected as a repeat monomer for the formation of a decamer. The succinate linker was employed when introducing the α-tocopherol head into the TPGS polymer. The final polymers were cross-linked and allowed to form amorphous cells. For the docking operation, however, PEG length depended on the maximum highest molecular weight accepted on the Maestro’s 2D sketcher platform. The P-gp protein was retrieved from Protein Data Bank (PDB ID: 6C0V) while PEG and TPGS chemical structures were downloaded from PubChem. The receptor and ligands were separately prepared using Glide’s protein preparation wizard and ligand preparation function, respectively, before the docking process. The Glide extra precision (XP) scoring mode, previously proven to effectively discriminate between P-gp substrates and inhibitors [61,62], was used for ligand-receptor docking in accordance with our previous study [63]. 

### 2.4. Phase 2: Synthesis and Optimization of the Sodium Alginate-Gelatine Bio-Ink as a 3D Printable Matrix for Nanoparticle Fixation

#### 2.4.1. Optimization of Printing Parameters

Several factors are important in producing the optimal printed tablet. The printing ink was optimized using various formulations of sodium alginate and gelatine under controlled conditions to determine the most accurate combination. The optimum printing parameters were obtained using various tests for printing head temperature, platform temperature, printing precision tip size, printing pressure, pre-flow delay, post-flow delay, the time between layers, and printing speed. The tests with results outlined are discussed in the following sections.

#### 2.4.2. Printability and Optimization of Sodium Alginate Gelatine Bio-Ink

Sodium alginate gel solutions of 3%, 5%, 8%, and 10% were prepared by mixing 100 mL of deionized ultrapure water with sodium alginate powder. The mixture was stirred on a magnetic stirrer for 1 h. The mixture was then placed in the fridge at 4 °C for 24 h to obtain a smooth homogenous gel. Gelatine gel solutions of 5%, 8%, and 10% were mixed in warm phosphate-buffered saline preheated to 60 °C on a magnetic stirrer for 30 min. The sodium alginate-gelatine printing ink was then prepared using 50 mL of warmed sodium alginate gel and 50 mL of warm gelatine mixture at different concentrations, the sodium alginate gelatine mixture was kept at a temperature of 60 °C. Previous studies have shown that maintaining gelatine gel at a stable high temperature before cooling increases strength and rigidity [64]. It was hypothesized that a higher concentration of gelatine increases the solidity of the devices after printing and before crosslinking in CaCl_2_. The combinations of mixtures were tested with higher concentrations of gelatine than sodium alginate.

#### 2.4.3. Optimum Hydrogel Printable Ink of Sodium Alginate/Gelatine

A 17 mm diameter × 3 mm (height) round template was designed to evaluate the printing accuracy of different concentrations of printing ink. The different concentration of bio-inks was loaded into a 30 mL plastic syringe. Three spherical tablet-shaped devices were printed with each bio-ink using a printing speed of 35.0 mm/s with a stainless steel 0.25 mm precision tip. The minimum pressure that continuous extrusion occurred was selected. Printed devices were measured with a caliper and the area of the devices was calculated as follows:

(1) $$A=\pi r^{2}$$

The printing accuracy was then determined by analyzing the actual area of the device, A_i_ (X mm^2^), against the designed area, *A* (227 mm^2^), using the following equation (Di Giuseppe et al., 2018):

(2) $$Printing\;Accuracy\;\left(\%\right)=[1-\left|\frac{A_{1}-A}{A}\right|]\;\times \;100$$

#### 2.4.4. Determination of the Effect of Needle Gauge on 3D Printing Accuracy

Multi-purpose stainless steel precision tip needles were used for 3D printing of the matrices. The optimized needle size for 3D printing of the bio-ink was determined by conducting printability tests using needles with a range of gauge sizes (G). Needle diameters used for printing were as follows (ID = inner diameter): 20 G (ID = 0.61 mm); 21 G (ID = 0.51 mm) 22 G (ID = 0.41 mm); 25 G (ID = 0.25 mm); 27 G (ID = 0.20 mm). Visual examination of the 3D printed strands was used to determine the optimal printing pressure for each needle size. The initial printing pressure was set at 500 kPa and the pressure during printing varied until a continuous uniform strand was printed. The minimum pressure that resulted in constant extrusion was then selected. The printing speed for each matrix was set at 30 mm/s. To evaluate the printing accuracy between the different needle sizes, a 15 × 15 × 3 mm square template was designed, and square-shaped matrices were printed using stainless-steel precision tips. The printed matrices were measured with a digital caliper and the matrix area was calculated using Equation (3).

(3) Volume=length×width

#### 2.4.5. Crosslinking of Sodium Alginate

**Figure 1:**
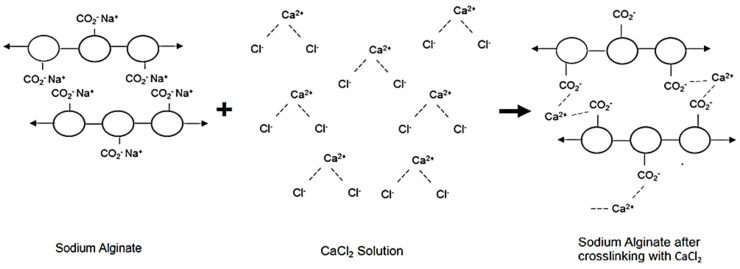
Sodium alginate gelation through crosslinking by exchange of sodium ions (Na^+^) and calcium ions (Ca^2+^).

Sodium alginate formed a crosslinked gel when added to a calcium chloride solution due to the cross-linker through the exchange of sodium ions (Na^+^) and calcium ions (Ca^2+)^. (Figure 1). 

#### 2.4.6. Temperature-Induced Crosslinking of Gelatine

Park Heon et al. reported the transition from solution to a gel to be 10% gelatine solution at 29 °C after cooling from 45 °C for approximately 5 min [65]. Time also plays a role in the gelation of a gelatine solution and as time increases, the gelatine solution is more likely to transition to gel at lower temperatures. Printing head temperatures were set at either 30, 35, or 40 °C (maximum head temperature 40 °C) to determine whether the printing ink’s temperature does not transition to gel within the cartridge during printing. Printing ink was loaded into a cartridge and used to print at 5, 10, 15, 20, 30, 45, 60, 90, and 120 min (min) through a 0.25 mm needle at a pressure of 2000 kPa until the ink gelation had occurred sufficiently to prevent extrusion. The printing needle was cleaned between each printing session to prevent possible clogging. 

#### 2.4.7. Determination of Optimum Printing Speed and Pre- and Post-Flow Delay Times

The most precise 3D printing of sodium alginate-gelatine nanoparticle formulation was at a printing speed of 25.0, 30.0, 35.0, and 40.0 mm/s at a pressure of 1.4 kPa bar with a stainless steel 0.25 mm precision tip. Pre- and post-flow delays were set at intervals of 0.01 s and printed layers were observed to determine the optimum pre- and post-flow delays, resulting in the layers not exhibiting any gaps.

### 2.5. Phase 3: Design and Synthesis of an Oral Chemotherapeutic 3D Printed Nanocomposite Hydrogel Tablet

#### 2.5.1. Preparation of Sodium Alginate-Gelatine Nanoparticle Formulation Printing Ink

The sodium alginate-gelatine (8% sodium alginate and 10% gelatine (*w*/*v*)) was stirred for 30 min at 60 °C. A polymeric combination of TPGS and PLGA containing 50 mg of MTX was dispersed into the 50 mL of sodium alginate-gelatine under constant stirring at 60 °C for 30 min to form nanocomposite hydrogels. The sodium alginate-gelatine nanoparticle formulation printing ink was formed. 

#### 2.5.2. Design of 3D Printed Tablets

Changing the shape and dimensions of a tablet can alter the way patients perceive medication and lead to greater willingness to adhere to treatment. Several studies have shown a link between the shape of oral medication tablets and the patient’s perception of the medication. Spherical tablets were preferred over angular tablets and were regarded as easier to swallow. Patient well-being can be increased by using the shape and color of pills to change how they perceive the overall sensory experience [66,67,68]. Therefore, the tablets in this study were designed to be disk-shaped tablets with a mean diameter of 15.0 mm and a height of 3.60 mm. 

The average volume of the tablets was calculated using the following formula:

(4) $$Volume=\pi r^{2\;}\times height$$

#### 2.5.3. Thermal Analysis of 3D Printed Tablets

Thermogravimetric analysis (TGA) was performed on the sodium alginate-gelatine nanoparticle formulation for oral drug delivery and blank sodium alginate-gelatine printing ink without nanoparticle formulations. Samples were heated at 10 °C/min in open aluminum pans, using nitrogen as a purge gas (25 mL/min). The percentage mass loss and/or onset temperature was calculated. 

#### 2.5.4. In Vitro Analysis of the 3D Printed Sodium Alginate-Gelatine Hydrogel-Nanoparticle Formulation for Oral Drug Delivery

Nanophotometer UV/Vis spectrophotometer was utilized to determine the maximum wavelength of an MTX and then plot the calibration curve to determine the release profile. In vitro, drug release studies were performed at both gastric and intestinal pH conditions. The 3D printed sodium alginate-gelatine nanoparticle formulation oral drug delivery devices were placed in 10 mL of simulated gastric (pH 1.2) and intestinal (pH 6.8) phosphate-buffered saline for a duration from 0 to 2.5 h. Samples were incubated during the release duration using an Orbit shaker incubator (LM-530-2, MRC Laboratory Instruments LTD., Hahistadrut, Holon, Israel) at 37 ± 0.5 °C and 30 rpm. Samples (0.5 mL) were withdrawn from simulated gastric release buffer every half an hour for 2.5 h simulated gastric samples, and at 30 min, then hourly for the first 12 h and at 24 h. The withdrawn liquid was replaced by a 0.1mL control solution at 37 °C for compensation. All extracted samples were filtered using a 0.45 Millipore Millex filter and stored at 4–8 °C. Ultraviolet-Visible spectroscopy was employed to determine the concentration of methotrexate. 

## 3. Results and Discussion

### 3.1. Phase 1: Synthesis of Methotrexate-Loaded Tocopheryl Polyethylene Glycol Succinate-Functionalized Polylactide-Co-Glycolic Acid Nanoparticles

#### 3.1.1. Assessment of MTX-Loaded TPGS-Functionalized PLGA Nanoparticles

The nanoprecipitation method used in this study to synthesize the MTX-loaded TPGS-functionalized NPs nanoparticles produced stable nano-constructs with desirable yields and unimodal particle distribution. The nanoparticle solution was collected as a white, free-flowing powder post lyophilization. Upon storage at 2 °C, the nanoparticle powder bed was easily reconstituted for further nanometric characterization. 

#### 3.1.2. Assessment of Nanoparticle Size and Zeta Potential

**Figure 2:**
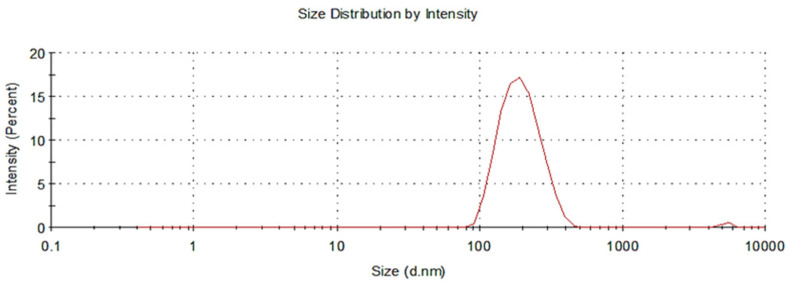
Particle size distribution profile of methotrexate-loaded polymeric TPGS-PLGA nanoparticles (nanoparticle formulation).

**Figure 3:**
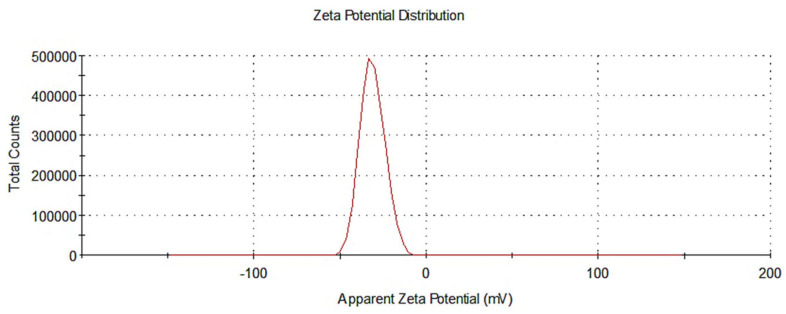
Distribution profile of zeta potential of the methotrexate-loaded TPGS-PLGA nanoparticles (nanoparticle formulation).

It has been shown that the cellular uptake of nanoparticles loaded with a drug is influenced by their shape and size. Nanoparticles with spherical shapes are significantly more likely to exhibit cellular binding and internalization than nanoparticles with different configurations. In this study, a nano-sized spherical nanoparticle (186.9 nm) with a particle size distribution (PDI) of 0.157, as demonstrated in Figure 2 was measured. Nanoparticles generally should be less than 150 nm to traverse the endothelial barrier; however, in cancer cells, the optimum nanoparticle size was found to be between 70 and 200 nm [69]. Therefore, the synthesized MTX-loaded TPGS-functionalized NPs were in a desirable range for the effective cellular uptake and internalization. In addition to size, a higher surface charge of particles prevents the aggregation of nanoparticles due to the increase in repellent energy. Zeta potential values of above ±30 mV have been proven to provide good stability of the nanoparticles in suspension with reduced aggregation and good dispersion. The zeta potential measured for the nanoparticle formulations in the current study was −31.4 mV, as seen in Figure 3. The higher surface charge of the nanoparticle formulations improved stability and reduced the risk of excessive aggregation and poor distribution. 

#### 3.1.3. Assessment of Nanoparticle Morphology

**Figure 4:**
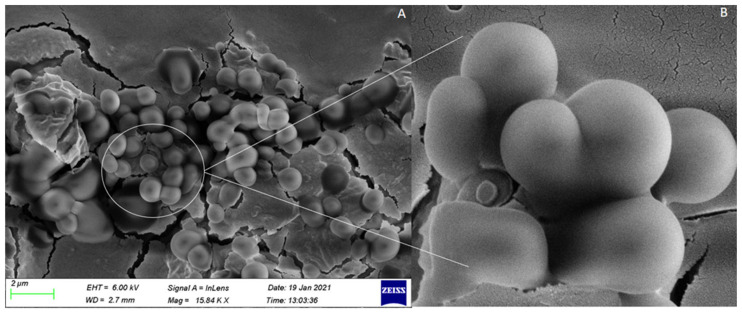
Gold-platinum sputtered SEM micrograph of methotrexate-loaded TPGS-PLGA nanoparticles. (**A**) Magnification =15.84 KX; Voltage=6.00 kV (**B**) Magnification=44.34 KX; Voltage=6.00  kV. SEM of optimized methotrexate loaded nanoparticle formulation showing sphericity, smooth surface of nanoparticles.

The polydispersity index (PDI) and zeta potential parameters were analyzed, and it was determined that the sample and molecule distribution was uniform. The average particle size and PDI were found to be less than 200 nm and 0.2, respectively. The size was found to be in an acceptable range for cellular uptake, and also PDI was within the optimum range of 0.1 ≤ PDI ≤ 0.5, which indicates the uniformity of the system [70]. The zeta potential values were -31.4 mV on average, indicating the colloidal stability of the system. The higher the surface net charge, the higher the repulsive forces to prevent particle aggregation during storage. The image in Figure 4 was captured by the scanning electron microscope (SEM), and the nanoparticle formulations were well distributed with spherical morphologies that had a size between 91 nm and 200 nm. The particle size established in the SEM was considerably aligned to those established in the zeta particle size analysis results.

#### 3.1.4. Thermogravimetric Analysis of MTX-Loaded TPGS-Functionalized PLGA Nanoparticles

**Figure 5:**
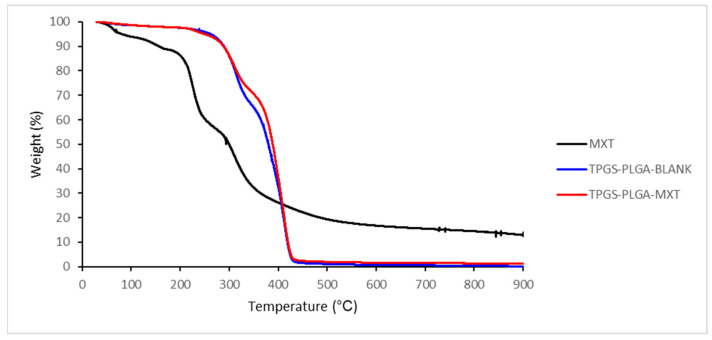
TGA curves of the methotrexate-loaded in polymeric TPGS-PLGA nanoparticles.

The TGA thermogram of unloaded TPGS-PLGA nanoparticles displayed a decomposition pattern, as shown in Figure 5. Initially, 30% of the weight was lost up to 350 °C, followed by a rapid decomposition at 430 °C, resulting in approximately 98% weight loss. Similarly, MTX-loaded TPGS-PLGA nanoparticles also exhibited two-stage weight loss. Initially, less than 30% of the weight was lost up to 350 °C, followed by a rapid decomposition with a weight loss of approximately 95% from a temperature of approximately 360 °C to 440 °C. The TGA of MTX showed a moderately different thermogram where there was a slight decomposition from 40 °C to 200 °C, approximately 15% weight loss. Then, followed by a rapid decomposition from approximately 210 °C to 250 °C, approximately 40% of the weight was lost, and the final decomposition took place at approximately 300 °C, with approximately 90% weight loss. This is also confirmed by the TGA thermogram reported by Dixit et al. [71].

#### 3.1.5. Differential Scanning Calorimetry Analysis of MTX-Loaded TPGS-Functionalized PLGA Nanoparticles

**Figure 6:**
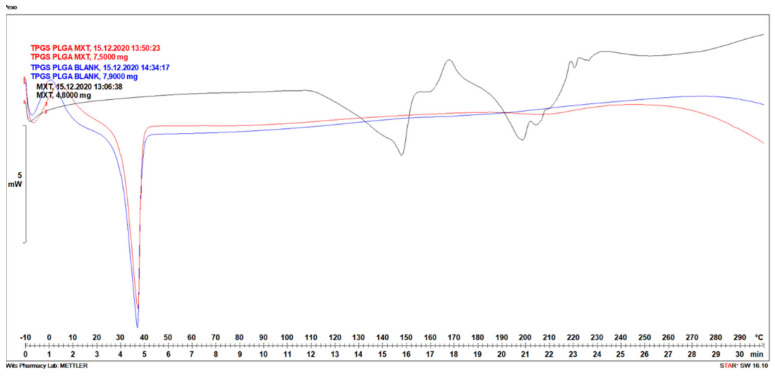
DSC curves of the methotrexate-loaded in polymeric TPGS- PLGA nanoparticles.

The TPGS-PLGA nanoparticles prepared were analyzed by DSC to investigate the crystal habit of MTX. Heat flow curves (DSC curves) depicted in Figure 6 for thermal characterization of methotrexate-loaded in polymeric TPGS-PLGA nanoparticles showed big endothermic peaks. Identifiable differences in melting temperatures of transitions were obtained. DSC thermogram of TPGS-PLGA nanoparticles (Blank) showed a sharp endothermic peak, the melting point at a temperature between 30 and 40 °C, as shown in Figure 6, this maybe be due to the low melting point of TPGS. The MTX melting temperature peak was noted, with broad endothermic peaks visible between 130–160 and 190–230 °C. This is also confirmed by the DSC thermogram reported by Pereira et al., 2013 [72]. MTX-loaded in polymeric TPGS-PLGA nanoparticles only showed a sharp endothermic peak at 30–40 °C for TPGS-PLGA, suggesting that MTX was transformed into an amorphous form. 

#### 3.1.6. Evaluation of the Chemical Stability and Integrity of the Various Nanosystem Components

**Figure 7:**
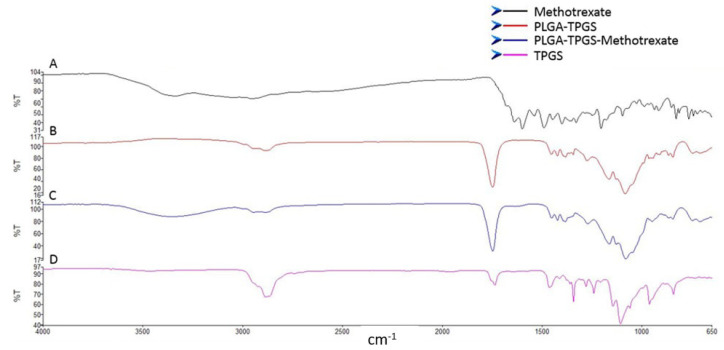
(**A**) FTIR spectra of methotrexate. (**B**) FTIR spectra of PLGA-TPGS. (**C**) FTIR spectra of methotrexate-loaded in polymeric TPGS-PLGA nanoparticles (nanoparticle formulation). (**D**) FTIR spectra of TPGS.

The FTIR spectra of PLGA, MTX, and the MTX-loaded TPGS-functionalized PLGA NPs are shown in Figure 7. MTX showed a typical waning of the broad signal from 3500 to 3000 cm^−1^ and sharpening of signals corresponding to O–H stretching from carboxyl groups and N–H stretching. Peaks at 1600–1670 cm^−1^ were linked to C=O stretching (-C=O stretching from the carboxylic group and C=O stretching from an amidic group) and 1400–1200 cm^−1^ corresponded to -C-O stretching from the carboxylic group, which is associated with -OH, C=C, and C-O functional groups of MTX (Figure 7A). All the bands identified in the FTIR spectrum are in accordance with the molecular structure of MTX [73]. The FTIR spectrum of PLGA-TPGS exhibited absorption peaks between 1000 and 1260 cm^−1^ related to C-O single bond extensions, and the broad absorption band at 2700 and 3000 cm^−1^ assigned to O-H stretching and implies the existence of free hydroxyl groups of PLGA (Figure 7B). The carbonyl band of TPGS appears at 1739 cm^−1^. The peaks seen at 1650–1100 cm^−1^ are attributed to O–H stretching vibrations as reported in the literature [74]. The FTIR spectrum of the MTX-loaded TPGS-functionalized PLGA NPs displayed harmonious peaks to PLGA and MTX (Figure 7C), with the NPs imbibing similar chemical composition, suggesting no significant chemical interaction between the functional groups of MTX and PLGA during the formation of the NPs. This is supported by similar studies in the literature [24]. 

#### 3.1.7. Drug Release Kinetics of Methotrexate from TPGS and PLGA Nanoparticles

**Figure 8:**
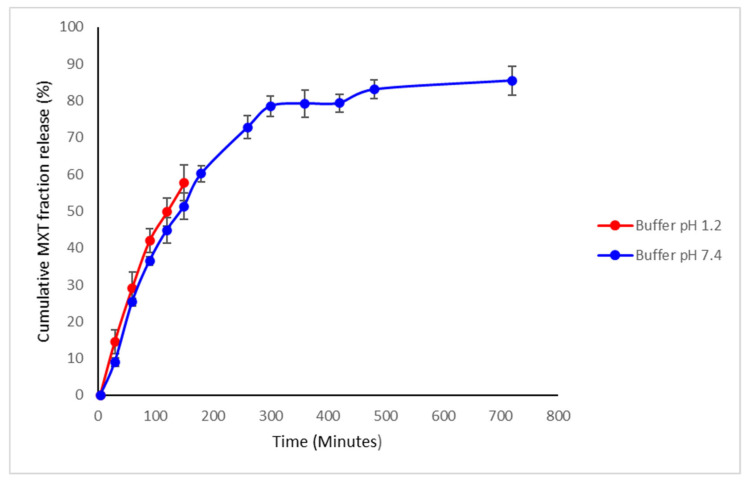
In vitro release of MTX from TPGS-PLGA nanoparticles at a stomach acidity of pH 1.2 and intestinal environment of pH 7.4 (*n* = 3, mean ± SD).

In vitro release of MTX from TPGS-PLGA nanoparticles was performed in a phosphate buffer system at pH 7.4 (PBS) and hydrochloric acid pH 1.2 (HCL), simulating the physiological and gastric pH conditions. MTX release from the TPGS-PLGA nanoparticles occurred in two phases, with an initial uniform rapid release of up to 80% within 5 h followed by a slow continuous cumulative fractional release phase over 24 h across the pH media of 7.4, as seen in Figure 8. The sample in a gastric (pH 1.2) simulated environment was sampled for up to 2.5 h because the administered content would have passed through the stomach at around this time. The MTX release pattern observed from the TPGS-PLGA nanoparticles indicates that the formulation is suitable for oral dosing.

There were no significant MTX release differences between pH 1.2 and 7.4 up to 2.5 h. The MTX was released up to 85% in 24 h in a simulated intestinal environment of pH 7.4.

### 3.2. Phase 2: Synthesis and Optimization of the Sodium Alginate-Gelatine Bio-Ink as a 3D Printable Matrix for Nanoparticle Fixation

#### 3.2.1. Optimization of Sodium Alginate/Gelatine Printing Ink

Three matrices of round devices were 3D printed with varying concentrations of bio-inks as listed in Table 1, using a printing speed of 35.0 mm/s with a stainless steel 0.25 mm precision tip. The minimum pressure at which constant extrusion occurs was selected. Table 1 shows the different combinations of sodium alginate and gelatine bio-printing ink prepared. The results revealed that the highest printing accuracy was obtained with 8% sodium alginate and 10% gelatine as the bio-gelatine ink.

**Figure 9:**
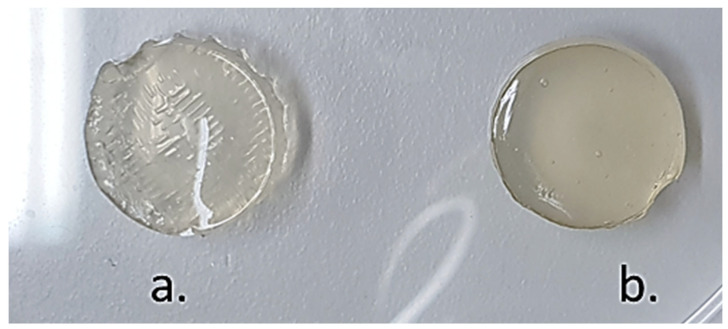
Images of typical 3D printed SA-GL tablet-shaped matrices produced using a 3D bioplotter in the least and most favorable concentrations: (**a**) Least favorable printing ink (5%SA-8%GL) and (**b**) Most accurate printing ink (8%SA-10%GL).

Increasing the concentration of SA resulted in higher printing accuracy, with bio-ink blends of 8% SA and 10% GL producing the best accuracy at 96.88%. The same was observed when the concentration of GL was increased, with no significant difference observed between concentrations of 5% and 8% GL (*p* > 0.05). Higher (10%) and lower (3%) concentrations of SA significantly reduced the printing accuracy for low (5%) and high (10%) concentrations of GL (Figure 9).

#### 3.2.2. The Influence of Printing Needle Size on 3D Printing Accuracy

The printing accuracy (%) of varying needle sizes was determined as described in Section 3.2.1 by analyzing the average area of three square 15 mm × 15 mm × 3 mm printed matrices per needle size. The results indicated that the highest printing accuracy (including a 15 s dwell time between layers) was achieved using a 25 G needle (Table 2).

#### 3.2.3. Optimization of Crosslinking Time of Sodium Alginate-Gelatine Devices in CaCl_2_

To further strengthen them after 3D printing, the tablets were transferred to a deep well petri dish and covered with 3 mL of 2% CaCl_2_ solution for 15 min. A study showed that crosslinking of the sodium alginate in the 3D printed devices significantly increases the hardness. The optimum crosslinking time determined was 15 min of immersion in CaCl_2_ [75]. The device was removed from the CaCl_2_ solution and placed in a petri dish to dry at 4 °C for 1 h. 

#### 3.2.4. Determination of Optimum Printing Temperature

At 30 °C, the printing ink was too thick to be extruded after 15 min. At 35 °C, the printing ink could be extruded for a maximum of 90 min. The printing ink remained fluid at 40 °C for more than 2 h, and printing was conducted with the printing headset at this temperature. The results are summarized in Table 3. 

The printing platform temperature was reduced to 0 °C since this was ideal to ensure rapid cooling of the printing ink once printed. Temperatures lower than 0 °C were found to stimulate premature crosslinking within the printing syringe during printing due to the proximity of the syringe to the printing platform. This resulted in the printing ink solidifying within the tip of the printing syringe and the inability of the printing ink to be extruded from the syringe. 

#### 3.2.5. The Influence of Printing Speed and Pre- and Post-Flow Dwell Times on 3D Printing Accuracy

Using a 15 × 15 mm square template and printing 3 layers, a 3D printed matrix per printing speed was produced using a printing speed of either 25 mm/s; 30 mm/s; 35 mm/s; 40 mm/s (max) as described in Section 3.2.1. The best printing speed was established as 35 mm/s (Table 4). A pre- and post-flow dwell time of 0.05 s was the most accurate to adjust appropriately for bio-ink flow lag.

#### 3.2.6. The Influence of Temperature on 3D Printing Accuracy

A bio-ink blend comprising 8% SA and 10% GL was loaded into a 3D printing cartridge and loaded into the 3D Bioplotter. Three layers of the bio-ink were printed using a 15 × 5 mm square template at 5, 10, 15, 20, 30, 45, 60, 90, and 120 min through a 0.25 mm needle at a pressure of 2000 kPa. This process was repeated three times (*n*= 3) for printing cartridges set at 30 °C, 35 °C, and 40 °C. The results are summarized in Table 4 and showed that at 30 °C, the bio-ink was too viscous to be extruded after 15 min. At 35 °C the bio-ink was extruded for a maximum of 90 min, and it remained fluid at 40 °C for >2 h. Therefore, all subsequent 3D printing was undertaken with the printing cartridge set to 40 °C. 

#### 3.2.7. Optimization of the Ionic Crosslinking Time of SA-GL Matrices

**Figure 10:**
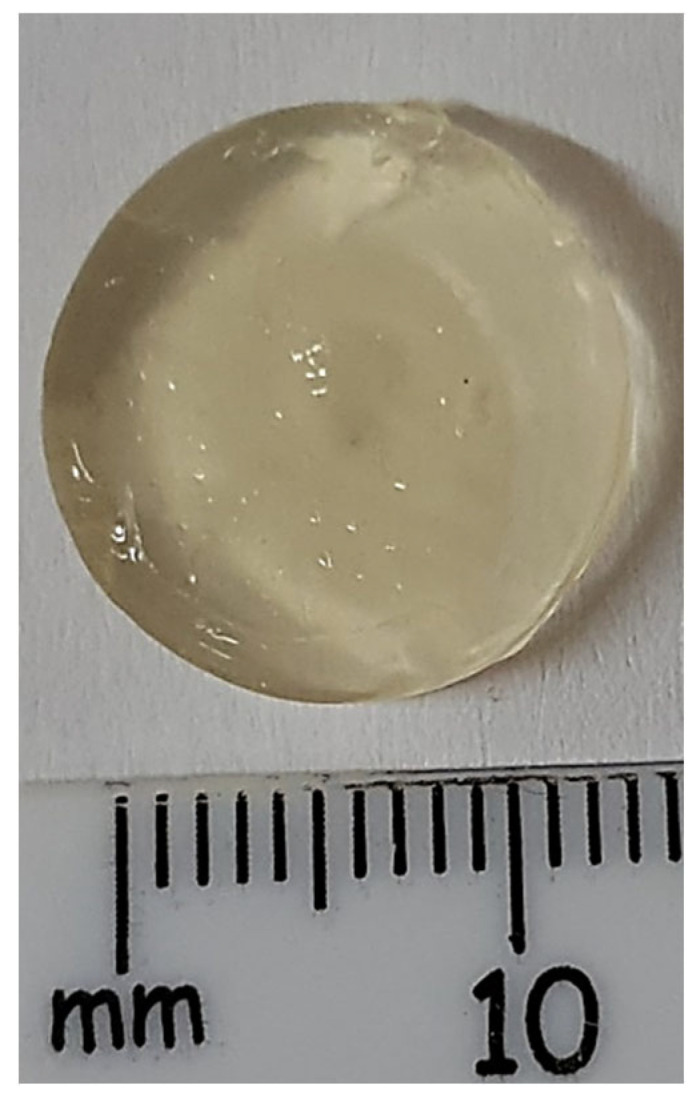
Dried 3D printed sodium alginate-gelatine hydrogel devices following temperature and chemical crosslinking in CaCl_2_.

The optimum crosslinking time was determined to be 15 min of immersion in CaCl_2_. The matrix was removed from the CaCl_2_ solution and placed in a petri dish to dry at 4 °C for 1 h. The resulting devices were uniform, round, and had good stability (Figure 10).

### 3.3. Phase 3: Design and Synthesis of an Oral Chemotherapeutic 3D Printed Nanocomposite Hydrogel Tablet

#### 3.3.1. The 3D Printing of Sodium Alginate-Gelatine Hydrogel Nanoparticle Formulation (Oral Chemotherapeutic Delivery System)

Sodium alginate-gelatine hydrogels were stirred and warmed at 60 °C, then the polymeric PLGA-TPGS nanoparticles loaded with MTX were incorporated to form a nanocomposite hydrogel system. The sodium alginate-gelatine hydrogel nanoparticle formulation for printing ink was then formed. Ten sodium alginate-gelatine hydrogel nanoparticle formulation tablets were printed using optimized printing parameters obtained (Figure 10). The printing platform was set to 0 °C to ensure that the gelatine temperature-induced hydrogen bond cross-linking occurs as soon as possible after printing, thereby increasing the stability of the structure [76,77]. The syringe temperature was set to 40 °C to prevent any cross-linking prior to printing. The tablet’s inner structure was formed by setting the 3D-Bioplotter to print at 90° angles at a printing distance of 0.3 mm and a printing speed of 35 mm/s. This ensured that the tablet had a solid infill. Layer height was set at 0.22 mm with a total of 13 layers printed. The time between layers was set at 15 s to allow adequate crosslinking to occur. 

After printing, sodium alginate-gelatine nanoparticle formulation tablets were placed in a round-bottom petri dish covered with a 2% calcium chloride solution for 30 min to facilitate crosslinking of the sodium alginate. Ten sodium alginate-gelatine nanoparticle formulation tablets were printed and had an average weight of 606 mg ± 9 mg. The sodium alginate-gelatine nanoparticle formulation dimensions had an average height of 3.56 mm and an average diameter of 14.98 mm. The average volume of the tablets was calculated as 626.83 mm^3^, and the tablets had an average drug load of 0.627 mg of MTX. 

#### 3.3.2. Determination of Thermal Stability of the Sodium Alginate-Gelatine Nanoparticle Formulation

**Figure 11:**
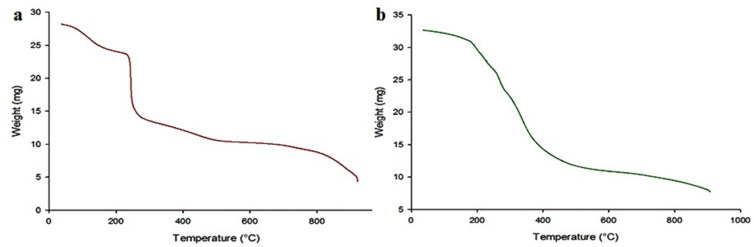
Thermogravimetric (TGA) analysis of, (**a**) sodium alginate-gelatine printing ink and (**b**) 3D printed sodium alginate-gelatine nanoparticle formulation.

TGA was used to distinguish and quantify the chemical or physical variations that occur upon heating a sample. TGA measures the quantity and degree of weight transformation of a substance in relation to a controlled temperature or time (Figure 11a,b). This information is then interpreted to predict the thermal stability of a substance and its components up to temperatures as high as 1000 °C by evaluating the weight changes caused by evaporation, dehydration, oxidation, and decomposition. Multiple stages of multiple weight loss phases may indicate the presence of several constituents in a substance [78]. The TGA curve shows that the sodium alginate-gelatine printing ink loses 14.8% of its weight at 200 °C. This initial weight loss may be due to evaporation of bound water.

Further 35.9% weight loss was observed between 200 °C and 280 °C. A further 13.1% weight loss was recorded between 280 °C and 600 °C, and the total weight loss value reached 79.4% at 900 °C. The TGA plot of the sodium alginate-gelatine nanoparticle formulation shows a slightly different trend compared to the sodium alginate-gelatine ink [79]. Reported a polymeric PLGA-TPGS to contribute to thermal degradation properties. A TGA analysis was performed on the synthesized PLGA-TPGS co-polymer to investigate its thermal properties. It was established that the sudden reduction in mass was between 215 °C and 300 °C [79]. In this study, the sodium alginate-gelatine nanoparticle formulation did not show this sudden reduction in weight between 240 °C and 260 °C. At 200 °C, 9.2% weight loss was observed and between 200 °C and 280 °C, there was a further weight loss of 18.3%. At the final temperature of 900 °C, the total weight loss reached was 75.6%. The results suggested that the sodium alginate-gelatine nanoparticle formulation had higher thermal stability due to the encapsulated drug-loaded nanoparticles.

#### 3.3.3. Drug Release Profiles of the Sodium Alginate-Gelatine Hydrogel Nanoparticulate System

To determine the ultraviolet (UV) absorbance of MTX, a solution of MTX in pH 7.4 phosphate-buffered saline with concentrations of 10–100 μg/mL was prepared. The concentration of the drug was measured against absorbance and a standard calibration curve was obtained with a linear equation as follows: y = 0.0004X + 0.0188 with an R^2^ of 0.9987. 

**Figure 12:**
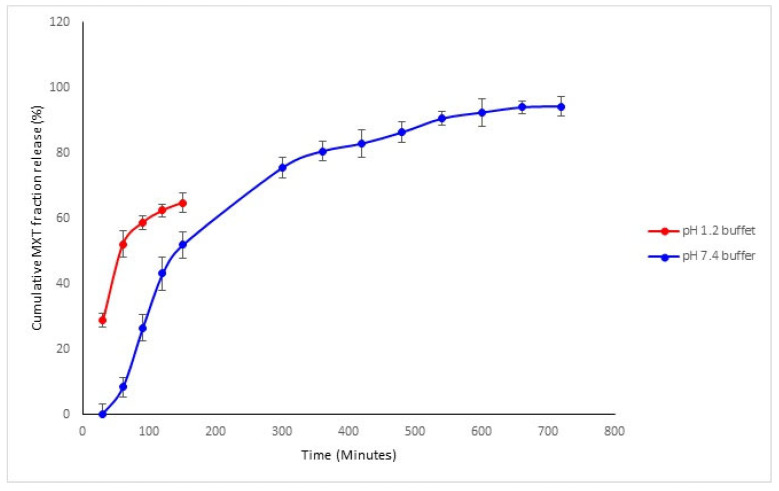
In vitro MTX release from sodium alginate-gelatine nanoparticle formulation conducted in pH 1.2 and pH 7.4 buffer (*n* = 3, mean ± SD).

In vitro release of sodium alginate-gelatine MTX-loaded nanoparticulate formulation was conducted at pH 7.4 and pH 1.2 to simulate the gastrointestinal environment, with sustained-release patterns in pH 7.4 buffer observed compared to pH 1.2 buffer (Figure 12). While at pH 1.2, the drug showed an initial burst release in the first 60 min, and then afterward, from 60 to 150 min, there was also a stable, sustained release of MTX (Figure 12). These results also indicate that the drug release was accelerated in a gastric acidic environment, validating that most of the MTX were released prior to entering the intestinal tract. It is therefore crucial that at this moment, P-gp is simultaneously inhibited to ensure that MTX is absorbed in sufficient concentrations. Hydrogels have characteristics that are similar to tissues but may suffer from burst release and rapid diffusion of chemotherapeutics from the polymer matrix due to pH sensitivity and swelling behavior. These gels can be investigated by monitoring the deflection of hydrogel-coated micro-cantilevers due to sensitive and repeatable responses to solution pH [80]. The hydrogel’s swelling was mainly due to chain relaxation of gelatine drug complexes caused by protonation of free amino groups in gelatine. The main advantage of hydrogels is the extent of drug release that can be controlled. In polymeric nanoparticles complexed within hydrogels, the drug release duration can be significantly prolonged, resulting in enhanced drug bioavailability [81]. 

### 3.4. Molecular Modelling of P-gp Inhibition by the TPGS

**Figure 13:**
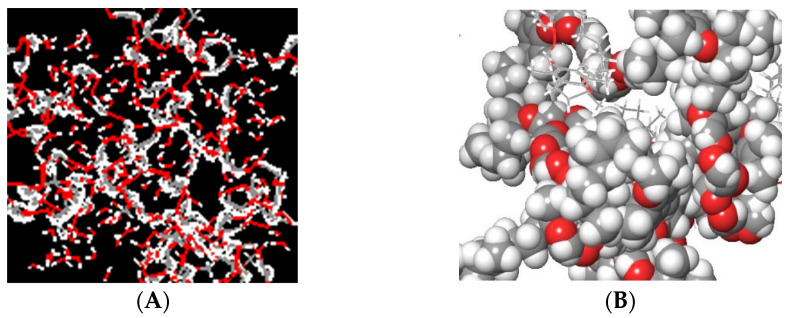
Polymeric structures of PEG (**A**) and TPGS (**B**). (**B**) shows the spherical 3D representation of the TPGS unit.

The PEG and TPGS polymer structures were successfully generated through repeating PEG monomers. While PEG was mainly hydrophilic (Figure 13A), the TPGS polymer featuring the succinate linker α-tocopherol tail imparted the hydrophobic character in the polymer (Figure 13B).

**Figure 14:**
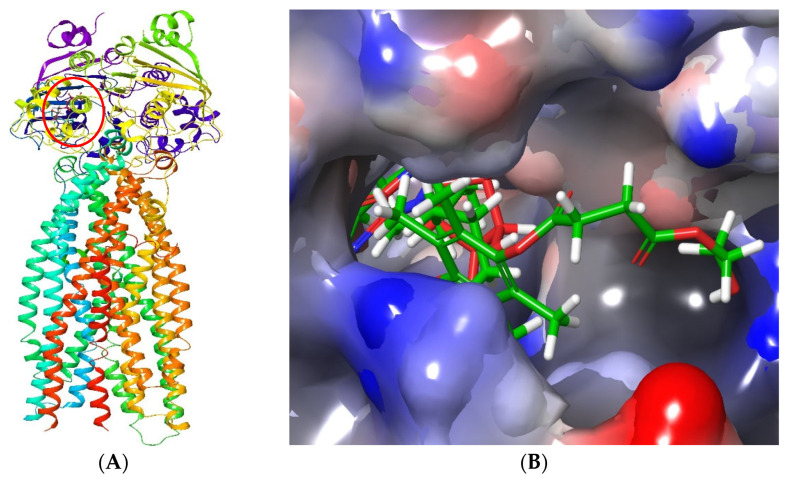
**The** 3D structures of the superimposed ATP and TPGS in complex with P-gp. (**A**) shows the location of the binding site (NBD) of ATP and TPGS (circled). (**B**) displays the superimposed ligands with ATP (red) confined into the deep binding cavity while TPGS (green), due to its polymeric nature, protrudes to the outside allosteric binding pocket with its hydrophobic α-tocopherol chain.

The model was validated by first docking ATP into the selected NBD site. This ligand retained the original binding pose and molecular interactions, including stabilization by the Mg^2+^ ion, with the binding site [54]. Moreover, the superimposition of TPGS and ATP complexed with P-gp confirmed that both ligands bind at the same site (Figure 14), in agreement with previous studies, however influenced by some degree of steric hindrance [50,51]. Figure 14, further reflects how the interaction of the hydrophobic chains, influences steric hindrance by the ATP-binding sites, where superimposed ligands with ATP are confined into the deep binding cavity as well. 

**Figure 15:**
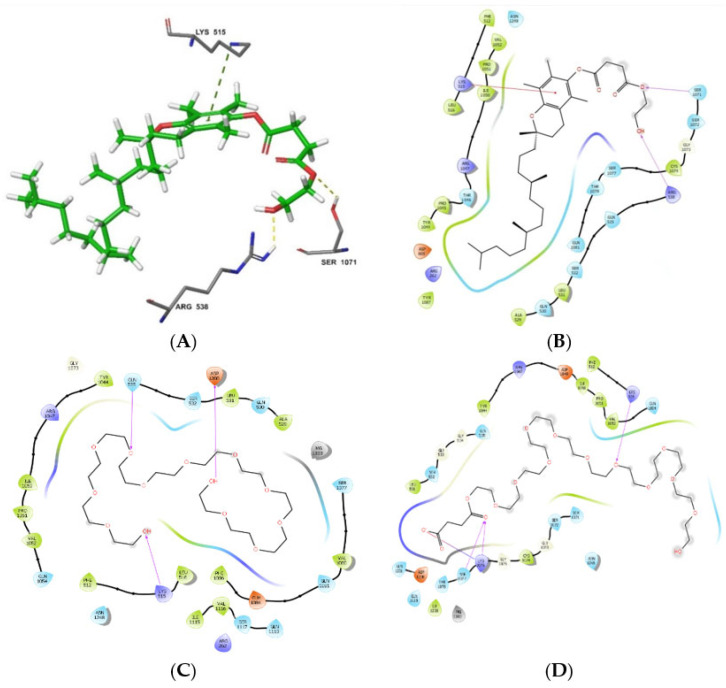
Molecular interactions of PEG and TPGS with P-gp ATP-binding site. (**A**) Shows predominant bonds stabilizing TPGS in a required pose and (**B**) highlights all amino acid residues in possible interaction with TPGS. (**C**,**D**) Respectively display the molecular interactions of PEG with PEG succinate with P-gp active site.

Consistent with the data reported by Liu et al. [51], TPGS interacted tightly with the binding site through its hydrophilic PEG tail. In this study, TPGS formed two hydrogen bonds with Arg^538^ and Ser^1071^ (PEG region) and was stabilized in position to assume the optimum crystal pose by Pi-interaction with Lys^515^ and its aromatic ring that forms part of the α-tocopherol, with a docking score of −7.849 (Figure 15A). The succinate portion that links PEG to the α-tocopherol played a great role in enforcing the required conformation for aromatic ring stabilization. The lipophilic α-tocopherol was involved in hydrophobic interactions with various amino acid residues, including Gln^1081^, Tyr^1086^, Thr^1078^, Arg^262^, Asp^805^, and Ala^529^ (Figure 15B). Additionally, this hydrophobic chain (Figure 14B, green ligand) protruded outside the deep ATP-binding cavity to the allosteric site, suggesting the ability to exert steric hindrance for the access of the binding site by ATP [53,82]. In fact, the α-tocopherol portion in TPGS has been reported to cause steric hindrance to the active site as well as block the allosteric P-gp site [18,50,53,83], while the PEG chain is potent in P-gp ATPase inhibition [84,85,86,87] and that modification of the PEG length alters ATPase inhibitory activity [50,83]. The results of this study suggest that the TPGS-based MTX nanoparticulate formulation results in increased bioavailability due to increased GI absorption afforded by the inhibition of P-gp by TPGS. 

In light of TPGS activity being mainly mediated through the PEG succinate portion, further docking was performed to intensely assess its interaction with P-gp. PEG with and without the succinate linker was docked to assess the impact of the succinate group in PEG binding. This study reports that the succinate linker plays a critical role in PEG binding to P-gp. Its positive effect was observed where the binding score of PEG (−8.139 kcal/mol) increased to −10.557 kcal/mol when it was incorporated. Furthermore, more favorable interactions, including an ionic bond with succinic acid Lys^1076^ as well as three hydrogen bonds with Lys^515^, Lys^1076^, and Ser^1077^ (Figure 15D), were established in comparison to the hydrogen bonding interactions with different amino acid residues such as Gln^353^ and Asp^1200^ in the absence of succinate (Figure 15C). Another key observation was the folded conformation of PEG in the absence of succinate (Figure 15C) but assuming an outstretched pose when linked to succinate (Figure 15D). This is consistent with the conformation assumed in the TPGS form (Figure 15B).

## 4. Conclusions

In phase 1 of this study, the MTX-loaded PLGA-TPGS polymeric nanoparticles were synthesized and characterized using FTIR. The morphology and size distribution of NPs were confirmed with SEM and Zetasizer, respectively. The nanoparticle formulations were well distributed and had a regular spherical shape between 91 nm and 200 nm in size, with a mean diameter of 186 nm. The NPs formulated demonstrated high stability with the zeta potential measured at −31.4 mV. MTX release kinetics from PLGA-TPGS polymeric nanoparticles were profiled successfully, with a release of up to 85% in the gastrointestinal environment. Following this, our molecular docking study showed the ATPase inhibition of P-gp by TPGS polymer, supporting enhanced GI absorption of MTX. The results above confirm the success of encapsulation and release of MTX by PLGA-TPGS NPs. In Phase 2 of our study, an optimized 3D printable bio-ink was developed with the most accurate 3D printing parameters for fabricating the SA-GL tablet-like matrices. Various combinations of SA-GL bio-inks were tested to determine the most efficient combination for 3D printing. The optimized 3D printing parameters with the highest printing accuracy value were obtained from a bio-ink comprising 8% SA and 10% GL. In Phase 3, the incorporation of PLGA-TPGS polymeric nanoparticles in alginate-gelatine hydrogels to form the oral drug delivery system was successfully performed and characterized. The results also indicated that the 3D printed hydrogel/nanocomposite system (including 15-sec pauses between layers) was achieved using a 25 G needle with an inner diameter of 25 mm, the optimal printing pressure was 1400 kPa, and 95% printing accuracy. Ten sodium alginate-gelatine nanoparticle formulations (oral drug delivery systems) were successfully printed with an average weight of 606 mg ± 9 mg. TGA analysis indicated that sodium alginate-gelatine nanoparticle formulations had a gradual loss of weight from 200 °C. Sodium alginate-gelatine nanoparticle formulation in buffer pH 6.8 demonstrated sustained release patterns while, at pH of 1.2, the drug showed an initial burst release in the first 60 min. This is an indication of a possible high GI absorption to improve drug bioavailability at the target site. Even though this study lacks an in vivo preclinical study, however, the effective encapsulation of PLGA-TPGS polymeric nanoparticles into alginate-gelatine hydrogel provides a good insight for future applications in controlled drug release systems.

## Figures and Tables

**Table 1:** Printing accuracy of different concentrations of sodium alginate/gelatine in printing ink.

SA-GL Blend	Printing Pressure (kPa)	Matrix Size (mm^2^)	Printing Accuracy (%)
3%SA-5%GL	800	175.12 ± 15.03	77.15 ± 6.62
5%SA-5%GL	800	197.74 ± 21.05	87.11 ± 9.27
5%SA-8%GL	900	247.23 ± 24.82	91.09 ± 8.91
8%SA-10%GL	1300	219.92 ± 5.44	96.88 ± 2.40
10%SA-10%GL	1500	250.65 ± 6.35	89.58 ± 2.80

**Table 2:** Assessment of 3D printing needle size and printing accuracy (%) of matrices.

Needle Gauge (G)	Needle Inner Diameter (mm)	Optimal Printing Pressure (kPa)	Printing Accuracy (%)
20	0.61	100	73.3
21	0.51	300	76.7
22	0.41	800	87.9
25	0.25	1400	95.6
27	0.20	2000	82.3

**Table 3:** Extrudability of printing ink at different temperatures.

Extrusion at	5 min	10 min	15 min	20 min	30 min	45 min	60 min	90 min	120 min
30 °C	✓	✓	✕	✕	✕	✕	✕	✕	✕
35 °C	✓	✓	✓	✓	✓	✓	✓	✕	✕
40 °C	✓	✓	✓	✓	✓	✓	✓	✓	✓

Keys: ✓ (achieved); ✕ (not achieved).

**Table 4:** Influence of 3D printing speed on printing accuracy.

Printing Speed (mm/s)	Printed Matrix Size (mm^2^)	Printing Accuracy (%)	Visual Inspection
25	N/A	N/A	Bio-ink was viscous and L2 resulted in printing needle extrusion into L3.
30	262	84	Bio-ink was over-extruded
35	227	99	Matrix was well-formed with no structural defects
40	212	94	Matrix had several structural defects
45	N/A	N/A	Matrix had several structural defects and was not capable of L formation

## Data Availability

The authors would like to acknowledge the Centre for High Performance Computing (CHPC) in collaboration with Council for Scientific and Industrial Research (CSIR) for providing the molecular docking license.

## References

[1] Abolmaali S.S., Tamaddon A.M., Dinarvand R. (2013). A review of therapeutic challenges and achievements of methotrexate delivery systems for treatment of cancer and rheumatoid arthritis. Cancer Chem. Phar..

[2] Zhao Y., Guo Y., Li R., Wang T., Han M., Zhu C., Wang X. (2016). Methotrexate nanoparticles prepared with codendrimer from polyamidoamine (PAMAM) and oligoethylene glycols (OEG) dendrons: Antitumor efficacy in vitro and in vivo. Sci. Rep..

[3] Gao X., Qian X.W., Zhu X.H., Yu Y., Miao H., Meng J.H., Jiang J.Y., Wang H.S., Zhai X.W. (2021). Population pharmacokinetics of high-dose methotrexate in Chinese pediatric patients with acute lymphoblastic leukemia. Front. Pharmacol..

[4] Bleyer W.A. (1978). The clinical pharmacology of methotrexate. New applications of an old drug. Cancer.

[5] Grim J., Chládek J., Martínková J. (2003). Pharmacokinetics and pharmacodynamics of methotrexate in non-neoplastic diseases. Clin. Pharm..

[6] Khan Z.A., Tripathi R., Mishra B. (2012). Methotrexate: A detailed review on drug delivery and clinical aspects. Expert Opin. Drug Deliv..

[7] Walvekar P., Gannimani R., Govender T. (2019). Combination drug therapy via nanocarriers against infectious diseases. Eur. J. Pharm. Sci..

[8] Teresi M.E., Crom W.R., Choi K.E., Mirro J., Evans W.E. (1987). Methotrexate bioavailability after oral and intramuscular administration in children. J. Pediatr..

[9] Amani A., Begdelo J.M., Yaghoubi H., Motallebinia S. (2019). Multifunctional magnetic nanoparticles for controlled release of anticancer drug, breast cancer cell targeting, MRI/fluorescence imaging, and anticancer drug delivery. J. Drug Del. Sci. Technol..

[10] Sanità G., Carrese B., Lamberti A. (2020). Nanoparticle Surface Functionalization: How to Improve Biocompatibility and Cellular Internalization. Front. Mol. Biosci..

[11] Jain A.K., Swarnakar N.K., Godugu C., Singh R.P., Jain S. (2011). The effect of the oral administration of polymeric nanoparticles on the efficacy and toxicity of tamoxifen. Biomaterials.

[12] Ensign L.M., Cone R., Hanes J. (2012). Oral drug delivery with polymeric nanoparticles: The gastrointestinal mucus barriers. Adv. Drug Deliv. Rev..

[13] Desai P.P., Date A.A., Patravale V.B. (2012). Overcoming poor oral bioavailability using nanoparticle formulations–opportunities and limitations. Drug Discov. Today Technol..

[14] Nanayakkara A.K., Follit C.A., Chen G., Williams N.S., Vogel P.D., Wise J.G. (2018). Targeted inhibitors of P-glycoprotein increase chemotherapeutic-induced mortality of multidrug resistant tumor cells. Sci. Rep..

[15] Gandhi S., Roy I. (2019). Doxorubicin-loaded casein nanoparticles for drug delivery: Preparation, characterization and in vitro evaluation. Intl. J. Biol. Macromol..

[16] Robey R.W., Pluchino K.M., Hall M.D., Fojo A.T., Bates S.E., Gottesman M.M. (2018). Revisiting the role of efflux pumps in multidrug-resistant cancer. Nat. Rev. Cancer.

[17] Guan Y., Wang L.Y., Wang B., Ding M.H., Bao Y.L., Tan S.W. (2020). Recent Advances of D-α-tocopherol Polyethylene Glycol 1000 Succinate Based Stimuli-responsive Nanomedicine for Cancer Treatment. Med. Sci..

[18] Tan S., Zou C., Zhang W., Yin M., Gao X., Tang Q. (2017). Recent developments in d-α-tocopheryl polyethylene glycol-succinate-based nanomedicine for cancer therapy. Drug Deliv..

[19] Yang C., Wu T., Qi Y., Zhang Z. (2018). Recent advances in the application of vitamin E TPGS for drug delivery. Theranostics.

[20] Saneja A., Khare V., Alam N., Dubey R.D., Gupta P.N. (2014). Advances in P-glycoprotein-based approaches for delivering anticancer drugs: Pharmacokinetic perspective and clinical relevance. Expert Opin. Drug Deliv..

[21] Essa D., Kondiah P.P., Choonara Y.E., Pillay V. (2020). The design of poly (lactide-co-glycolide) nanocarriers for medical applications. Front. Bioeng. Biotechnol..

[22] Makadia H.K., Siegel S.J. (2011). Poly lactic-co-glycolic acid (PLGA) as biodegradable controlled drug delivery carrier. Polymers.

[23] Rezvantalab S., Drude N.I., Moraveji M.K., Güvener N., Koons E.K., Shi Y., Lammers T., Kiessling F. (2018). PLGA-Based Nanoparticles in Cancer Treatment. Front. Pharmacol..

[24] Maleki H., Dorkoosh F., Adabi M., Khosravani M., Arzani H., Kamali M. (2017). Methotrexate-loaded plga nanoparticles: Preparation, characterization and their cytotoxicity effect on human glioblastoma U87MG cells. Int. J. Med. Nano Res..

[25] Tan K.X., Danquah M.K., Sidhu A., Ongkudon C.M., Lau S.Y. (2017). Towards targeted cancer therapy: Aptamer or oncolytic virus?. Eur. J. Pharm. Sci..

[26] Yang Y.Y., Chung T.S., Ng N.P. (2001). Morphology, drug distribution, and in vitro release profiles of biodegradable polymeric microspheres containing protein fabricated by double-emulsion solvent extraction/evaporation method. Biomaterials.

[27] Lu B., Lv X., Le Y. (2019). Chitosan-modified PLGA nanoparticles for control-released drug delivery. Polymers.

[28] Moroz E., Matoori S., Leroux J.-C. (2016). Oral delivery of macromolecular drugs: Where we are after almost 100 years of attempts. Adv. Drug Deliv. Rev..

[29] Cerchiara T., Abruzzo A., Parolin C., Vitali B., Bigucci F., Gallucci M.C., Nicoletta F.P., Luppi B. (2016). Microparticles based on chitosan/carboxymethylcellulose polyelectrolyte complexes for colon delivery of vancomycin. Carbohydr. Polym..

[30] Rizwan M., Yahya R., Hassan A., Yar M., Azzahari A.D., Selvanathan V., Sonsudin F., Abouloula C.N. (2017). pH sensitive hydrogels in drug delivery: Brief history, properties, swelling, and release mechanism, material selection and applications. Polymers.

[31] Ferreira N.N., Perez T.A., Pedreiro L.N., Prezotti F.G., Boni F.I., Cardoso V.M.D.O., Venâncio T., Gremião M.P.D. (2017). A novel pH-responsive hydrogel-based on calcium alginate engineered by the previous formation of polyelectrolyte complexes (PECs) intended to vaginal administration. Drug Dev. Ind. Pharm..

[32] Mikolaszek B., Kazlauske J., Larsson A., Sznitowska M. (2020). Controlled Drug Release by the Pore Structure in Polydimethylsiloxane Transdermal Patches. Polymers.

[33] Schütz K., Placht A.M., Paul B., Brüggemeier S., Gelinsky M., Lode A. (2017). Three-dimensional plotting of a cell-laden alginate/methylcellulose blend: Towards biofabrication of tissue engineering constructs with clinically relevant dimensions. J. Tissue Eng. Regen. Med..

[34] Pietrzak K., Isreb A., Alhnan M.A. (2015). A flexible-dose dispenser for immediate and extended release 3D printed tablets. Eur. J. Pharm. Biopharm..

[35] Shin J.H., Kang H.-W. (2018). The development of gelatin-based bio-ink for use in 3D hybrid bioprinting. Int. J. Precis Eng. Manuf..

[36] Wang X., Ao Q., Tian X., Fan J., Tong H., Hou W., Bai S. (2017). Gelatin-based hydrogels for organ 3D bioprinting. Polymers.

[37] Isoda T., Ito S., Kajiwara M., Nagasawa M. (2007). Successful high-dose methotrexate chemotherapy in a patient with acute lymphocytic leukemia who developed acute renal failure during the initial treatment. Pediatr. Int..

[38] Cowan D.S., Tannock I.F. (2001). Factors that influence the penetration of methotrexate through solid tissue. Int. J. Cancer.

[39] Alkholief M., Albasit H., Alhowyan A., Alshehri S., Raish M., Kalam M.A., Alshamsan A. (2019). Employing a PLGA-TPGS based nanoparticle to improve the ocular delivery of Acyclovir. Saudi Pharm. J..

[40] Gaonkar R.H., Ganguly S., Dewanjee S., Sinha S., Gupta A., Ganguly S., Chattopadhyay D., Chatterjee Debnath M. (2017). Garcinol loaded vitamin E TPGS emulsified PLGA nanoparticles: Preparation, physicochemical characterization, in vitro and in vivo studies. Sci. Rep..

[41] Sardo H.S., Saremnejad F., Bagheri S., Akhgari A., Garekani H.A., Sadeghi F. (2019). A review on 5-aminosalicylic acid colon-targeted oral drug delivery systems. Int. J. Pharm..

[42] Cerqueira B.B.S., Lasham A., Shelling A.N., Al-Kassas R. (2017). Development of biodegradable PLGA nanoparticles surface engineered with hyaluronic acid for targeted delivery of paclitaxel to triple negative breast cancer cells. Mater. Sci. Eng. C.

[43] Lawson G., Ogwu J., Tanna S. (2018). Quantitative screening of the pharmaceutical ingredient for the rapid identification of substandard and falsified medicines using reflectance infrared spectroscopy. PLoS ONE.

[44] Hunter J., Jepson M.A., Tsuruo T., Simmons N.L., Hirst B.H. (1993). Functional expression of P-glycoprotein in apical membranes of human intestinal Caco-2 cells. Kinetics of vinblastine secretion and interaction with modulators. J. Biol. Chem..

[45] Yano K., Tomono T., Ogihara T. (2018). Advances in studies of P-glycoprotein and its expression regulators. Biol. Pharm. Bull..

[46] Drozdzik M., Czekawy I., Oswald S., Drozdzik A. (2020). Intestinal drug transporters in pathological states: An overview. Pharmacol. Rep..

[47] Mora Lagares L., Minovski N., Caballero Alfonso A.Y., Benfenati E., Wellens S., Culot M., Gosselet F., Novič M. (2020). Homology Modeling of the Human P-glycoprotein (ABCB1) and Insights into Ligand Binding through Molecular Docking Studies. Int. J. Mol. Sci..

[48] Sharom F.J. (2011). The P-glycoprotein multidrug transporter. Essays Biochem..

[49] Prajapati R., Sangamwar A.T. (2014). Translocation mechanism of P-glycoprotein and conformational changes occurring at drug-binding site: Insights from multi-targeted molecular dynamics. Biochim. Biophys. Acta (BBA)—Biomembr..

[50] Collnot E.M., Baldes C., Wempe M.F., Kappl R., Hüttermann J., Hyatt J.A., Edgar K.J., Schaefer U.F., Lehr C.M. (2007). Mechanism of Inhibition of P-Glycoprotein Mediated Efflux by Vitamin E TPGS:  Influence on ATPase Activity and Membrane Fluidity. Mol. Pharm..

[51] Liu T., Liu X., Xiong H., Xu C., Yao J., Zhu X., Zhou J., Yao J. (2018). Mechanisms of TPGS and its derivatives inhibiting P-glycoprotein efflux pump and application for reversing multidrug resistance in hepatocellular carcinoma. Polym. Chem..

[52] Bogman K., Erne-Brand F., Alsenz J., Drewe J. (2003). The role of surfactants in the reversal of active transport mediated by multidrug resistance proteins. J. Pharm. Sci..

[53] Luiz M.T., Di Filippo L.D., Alves R.C., Araújo V.H.S., Duarte J.L., Marchetti J.M., Chorilli M. (2021). The use of TPGS in drug delivery systems to overcome biological barriers. Eur. Polym. J..

[54] Kim Y., Chen J. (2018). Molecular structure of human P-glycoprotein in the ATP-bound, outward-facing conformation. Science.

[55] Alam A., Kowal J., Broude E., Roninson I., Locher K.P. (2019). Structural insight into substrate and inhibitor discrimination by human P-glycoprotein. Science.

[56] Urgaonkar S., Nosol K., Said A.M., Nasief N.N., Bu Y., Locher K.P., Lau J.Y., Smolinski M.P. (2022). Discovery and characterization of potent dual P-glycoprotein and CYP3A4 inhibitors: Design, synthesis, cryo-EM analysis, and biological evaluations. J. Med. Chem..

[57] Chen L., Li Y., Yu H., Zhang L., Hou T. (2012). Computational models for predicting substrates or inhibitors of P-glycoprotein. Drug Discov. Today.

[58] Shahbaaz M., Nkaule A., Christoffels A. (2019). Designing novel possible kinase inhibitor derivatives as therapeutics against Mycobacterium tuberculosis: An in silico study. Sci. Rep..

[59] Kryscio D.R., Shi Y., Ren P., Peppas N.A. (2011). Molecular docking simulations for macromolecularly imprinted polymers. Ind. Eng. Chem. Res..

[60] Crouch E.C., Smith K., McDonald B., Briner D., Linders B., McDonald J., Holmskov U., Head J., Hartshorn K. (2006). Species differences in the carbohydrate binding preferences of surfactant protein D. Am. J. Respir. Cell Mol. Biol..

[61] Dolghih E., Bryant C., Renslo A.R., Jacobson M.P. (2011). Predicting binding to p-glycoprotein by flexible receptor docking. PLoS Comput. Biol..

[62] Friesner R.A., Murphy R.B., Repasky M.P., Frye L.L., Greenwood J.R., Halgren T.A., Sanschagrin P.C., Mainz D.T. (2006). Extra Precision Glide:  Docking and scoring incorporating a model of hydrophobic enclosure for protein−ligand complexes. J. Med. Chem..

[63] Rants’o T.A., van der Westhuizen C.J., van Zyl R.L. (2022). Optimization of covalent docking for organophosphates interaction with Anopheles acetylcholinesterase. J. Mol. Graph. Model..

[64] Fonkwe L.G., Narsimhan G., Cha A.S. (2003). Characterization of gelation time and texture of gelatin and gelatin–polysaccharide mixed gels. Food Hydrocoll..

[65] Park H.E., Gasek N., Hwang J., Weiss D.J., Lee P.C. (2020). Effect of temperature on gelation and cross-linking of gelatin methacryloyl for biomedical applications. Phys. Fluids.

[66] Wan X., Woods A.T., Salgado-Montejo A., Velasco C., Spence C. (2015). Assessing the expectations associated with pharmaceutical pill colour and shape. Food Qual. Prefer..

[67] Lajoinie A., Henin E., Nguyen K.A., Malik S., Mimouni Y., Sapori J.M., Bréant V., Cochat P., Kassai B. (2016). Oral drug dosage forms administered to hospitalized children: Analysis of 117,665 oral administrations in a French paediatric hospital over a 1-year period. Int. J. Pharm..

[68] Ternik R., Liu F., Bartlett J.A., Khong Y.M., Tan D.C.T., Dixit T., Wang S., Galella E.A., Gao Z., Klein S. (2018). Assessment of swallowability and palatability of oral dosage forms in children: Report from an M-CERSI pediatric formulation workshop. Int. J. Pharm..

[69] Gaumet M., Vargas A., Gurny R., Delie F. (2008). Nanoparticles for drug delivery: The need for precision in reporting particle size parameters. Eur. J. Pharm. Biopharm..

[70] Shah R., Eldridge D., Palombo E., Harding I. (2014). Optimisation and stability assessment of solid lipid nanoparticles using particle size and zeta potential. J. Phys. Sci..

[71] Dixit A., Kulkarni P., Redyy S. (2014). Methotrexate fast disintegrating tablet as a dosage form for dysphagia patients. Int. J. Pharm. Pharm. Sci..

[72] Pereira A.D.F., Pereira L.G.R., Barbosa L.A.D.O., Fialho S.L., Pereira B.G., Patricio P.S.D.O., Pinto F.C.H., Da Silva G.R. (2013). Efficacy of methotrexate-loaded poly (ε-caprolactone) implants in Ehrlich solid tumor-bearing mice. Drug Deliv..

[73] Fuliaş A., Popoiu C., Vlase G., Vlase T., Oneţiu D., Săvoiu G., Simu G., Pătruţescu C., Ilia G., Ledeţi I. (2014). Thermoanalytical and spectroscopic study on methotrexate–active substance and tablet. Dig. J. Nanomater. Biostruct.

[74] Şimşek S., Eroğlu H., Kurum B., Ulubayram K. (2013). Brain targeting of Atorvastatin loaded amphiphilic PLGA-b-PEG nanoparticles. J. Microencapsul..

[75] Di Giuseppe M., Law N., Webb B., Macrae R.A., Liew L.J., Sercombe T.B., Dilley R.J., Doyle B.J. (2018). Mechanical behaviour of alginate-gelatin hydrogels for 3D bioprinting. J. Mech. Behav. Biomed. Mater..

[76] Zhao Y., Li Y., Mao S., Sun W., Yao R. (2015). The influence of printing parameters on cell survival rate and printability in microextrusion-based 3D cell printing technology. Biofabrication.

[77] Xing Q., Yates K., Vogt C., Qian Z., Frost M.C., Zhao F. (2014). Increasing mechanical strength of gelatin hydrogels by divalent metal ion removal. Sci. Rep..

[78] Stodghill S.P. (2010). THERMAL ANALYSIS: Thermal analysis—A review of techniques and applications in the pharmaceutical sciences. Am. Pharm. Rev..

[79] Ma Y., Zheng Y., Liu K., Tian G., Tian Y., Xu L., Yan F., Huang L., Mei L. (2010). Nanoparticles of poly (lactide-co-glycolide)-da-tocopheryl polyethylene glycol 1000 succinate random copolymer for cancer treatment. Nanoscale Res. Lett..

[80] Mao J., Kondu S., Ji H.F., McShane M.J. (2006). Study of the near-neutral pH-sensitivity of chitosan/gelatin hydrogels by turbidimetry and microcantilever deflection. Biotechnol. Bioeng..

[81] Sharpe L.A., Daily A.M., Horava S.D., Peppas N.A. (2014). Therapeutic applications of hydrogels in oral drug delivery. Expert Opin. Drug Deliv..

[82] Batrakova E.V., Li S., Li Y., Alakhov V.Y., Kabanov A.V. (2004). Effect of pluronic P85 on ATPase activity of drug efflux transporters. Pharm. Res..

[83] Collnot E.M., Baldes C., Schaefer U.F., Edgar K.J., Wempe M.F., Lehr C.M. (2010). Vitamin E TPGS P-Glycoprotein Inhibition Mechanism: Influence on Conformational Flexibility, Intracellular ATP Levels, and Role of Time and Site of Access. Mol. Pharm..

[84] Johnson B.M., Charman W.N., Porter C.J. (2002). An in vitro examination of the impact of polyethylene glycol 400, Pluronic P85, and vitamin E d-alpha-tocopheryl polyethylene glycol 1000 succinate on P-glycoprotein efflux and enterocyte-based metabolism in excised rat intestine. AAPS PharmSci.

[85] Shen Q., Lin Y., Handa T., Doi M., Sugie M., Wakayama K., Okada N., Fujita T., Yamamoto A. (2006). Modulation of intestinal P-glycoprotein function by polyethylene glycols and their derivatives by in vitro transport and in situ absorption studies. Int. J. Pharm..

[86] Hugger E.D., Novak B.L., Burton P.S., Audus K.L., Borchardt R.T. (2002). A comparison of commonly used polyethoxylated pharmaceutical excipients on their ability to inhibit P-glycoprotein activity in vitro. J. Pharm. Sci..

[87] Gurjar R., Chan C.Y., Curley P., Sharp J., Chiong J., Rannard S., Siccardi M., Owen A. (2018). Inhibitory effects of commonly used excipients on P-Glycoprotein in vitro. Mol. Pharm..

